# Genome-wide investigation of the heat shock transcription factor (*Hsf*) gene family in Tartary buckwheat (*Fagopyrum tataricum*)

**DOI:** 10.1186/s12864-019-6205-0

**Published:** 2019-11-15

**Authors:** Moyang Liu, Qin Huang, Wenjun Sun, Zhaotang Ma, Li Huang, Qi Wu, Zizhong Tang, Tongliang Bu, Chenglei Li, Hui Chen

**Affiliations:** 1 College of Life Science, Sichuan Agricultural University, Ya’an, China; 2 School of Agriculture and Biolog, Shanghai Jiao Tong University, Shanghai, China

**Keywords:** Tartary buckwheat, *FtHsf* genes, Genome-wide, Expression patterns, Evolution

## Abstract

**Background:**

Heat shock transcription factor (Hsfs) is widely found in eukaryotes and prokaryotes. Hsfs can not only help organisms resist high temperature, but also participate in the regulation of plant growth and development (such as involved in the regulation of seed maturity and affects the root length of plants). The *Hsf* gene was first isolated from yeast and then gradually found in plants and sequenced, such as *Arabidopsis thaliana*, rice, maize. Tartary buckwheat is a rutin-rich crop, and its nutritional value and medicinal value are receiving more and more attention. However, there are few studies on the *Hsf* genes in Tartary buckwheat. With the whole genome sequence of Tartary buckwheat, we can effectively study the *Hsf* gene family in Tartary buckwheat.

**Results:**

According to the study, 29 *Hsf* genes of Tartary buckwheat *(FtHsf)* were identified and renamed according to location of *FtHsf* genes on chromosome after removing a redundant gene. Therefore, only 29 *FtHsf* genes truly had the functional characteristics of the *FtHsf* family. The 29 *FtHsf* genes were located on 8 chromosomes of Tartary buckwheat, and we found gene duplication events in the *FtHsf* gene family, which may promote the expansion of the *FtHsf* gene family. Then, the motif compositions and the evolutionary relationship of FtHsf proteins and the gene structures, cis-acting elements in the promoter, synteny analysis of *FtHsf* genes were discussed in detail. What’s more, we found that the transcription levels of *FtHsf* in different tissues and fruit development stages were significantly different by quantitative real-time PCR (qRT-PCR), implied that *FtHsf* may differ in function.

**Conclusions:**

In this study, only 29 *Hsf* genes were identified in Tartary buckwheat. Meanwhile, we also classified the *FtHsf* genes, and studied their structure, evolutionary relationship and the expression pattern. This series of studies has certain reference value for the study of the specific functional characteristics of Tartary buckwheat *Hsf* genes and to improve the yield and quality of Tartary buckwheat in the future.

## Background

High temperature affects the growth, development and metabolism of plants [1–4]. Heat shock transcription factors are the main regulator of heat stress response, and it is important for eukaryotes and prokaryotes to resist high temperature [5–8]. When in a hot environment, *Hsfs* activate heat shock proteins (Hsps) by binding to the heat stress elements (HSEs) in *Hsps* promoter to resist high temperature [7, 9–14]. There is a ubiquitous heat shock response mechanism in plants, which includes a series of complex reactions, such as new protein synthesis, folding, specific biological functions and so on. In these proteins, Hsps as molecular chaperones, are essential to maintaining or restoring protein homeostasis [15–19].

A typical Hsf protein contains five domains, including a DNA-binding domain (DBD), an oligomerization domain (OD) or hydrophobic repeat domain (HR-A/B) [20, 21], a nuclear localization signal domain (NLS), a nuclear export signal domain (NES) and an activator motif (AHA) [20, 22, 23]. Because of the differences in the HR-A/B domain of Hsf family members, the *Hsf* genes are divided into three big groups, named A (from A1 to A10), B (from B1 to B4) and C (from C1 to C2). It is worth noting that there is a AHA region which only exists in some members of group A, and the AHA region is the key area for Hsfs to play a self-activating role [21, 24].

Tartary buckwheat is a widely cultivated dicotyledonous nutritious food crop. Tartary buckwheat fruit contains abundant and balanced essential amino acids, and its total protein content is richer than that of main grain crops [25–28]. The *Hsfs* not only play a key role in plants resistance to high temperatures and improvements of plants heat tolerance, but also can regulate the growth and development of plants [29]. The *Hsf* genes family have been studied in many plants, and these studies were based on the heat stress response of Hsfs [22, 30, 31], but there were few studies on the regulation of plant growth and development by *Hsfs*. Because of the important role of *Hsf* genes in various phylogenetic and its resistance to high temperature (such as involved in the regulation of seed maturity and affects the root length of plants [5, 32]), it is of great significance to have a detailed study on the Tartary buckwheat *Hsf* gene family. Thanks to the complete genome sequencing of Tartary buckwheat, we can systematically research the *Hsf* gene family on the whole genome level. In this study, we firstly introduced the gene structures, cis-acting elements in the promoter, chromosomal locations, homology analysis, expression patterns of 29 Tartary buckwheat *Hsf* genes and motif compositions and phylogenetic analysis of 29 Tartary buckwheat Hsf proteins in detail. Secondly, the synteny analysis and phylogenetic relationships of *Hsf* genes between *Fagopyrum tataricum* and *Beta vulgaris, Glycine max, Helianthus annuus, Oryza sativa, Solanum lycopersicum, Vitis vinifera, Arabidopsis thaliana* were compared. Then, the expression patterns of the *Hsf* genes in different tissues were determined by qRT-PCR. More importantly, we also measured the transcriptional level of *Hsf* genes during fruit development. To sum up, this research provides valuable clues for studying the action mechanism of some members of the *FtHsf* gene family during buckwheat growth and development.

## Methods

### Plant growth

XIQIAO is one of buckwheat varieties, and it is rich in rutin. Since 2013, XIQIAO has grown under the same experimental conditions in the experimental base locate at the farm, Sichuan Agricultural University [33]. As for the experimental samples, we collected the materials including the fruits from three different stages (13, 19, and 25 days after pollination, DAP), the flowers, the stems, the roots, and the leaves from five strains of Tartary buckwheat in the same physiological state [34]. The collected samples were stored in − 80 °C refrigerator for subsequent study.

### Genes identification

The genome sequence of Tartary buckwheat genome was obtained from the Tartary Buckwheat Genome Project. Firstly, the candidate Hsf proteins of Tartary buckwheat were authenticated by a BLASTp search. Then, we downloaded the Hsf domain (PF00447) from the Pfam database. According to the HMMER3, we used this date to build a HMM file. Finally, Hsf proteins were used as initial queries on the NCBI protein database (https://blast.ncbi.nlm.nih.gov/Blast.cgi? PROGRAM = blastp&PAGE_TYPE = BlastSearch&LINK_LOC = blasthome) by BLASTp, further verifying that Hsf proteins derived from Tartary buckwheat belong to the *Hsf* gene family. The results showed that 29 *Hsf* genes were identified as heat transcription factors of Tartary buckwheat. Besides, the isoelectric point, sequence length and molecular weight were acquired through the ExPasy (https://web.expasy.org/protparam/), and the subcellular localization of the Hsf proteins identified were obtained using CELLO (http://cello.life.nctu.edu.tw/) (Additional file 1).

### Phylogenetic analysis

The Hsfs of *Arabidopsis thaliana* and the Hsfs of Tartary buckwheat were constructed into a phylogenetic tree by Neighbor-Joining (NJ) method, and all Hsfs were divided into three big groups. In addition, we constructed a multi-species phylogenetic evolutionary tree including FtHsf protein sequences and *Vitis vinifera*, *Solanum lycopersicum*, *Oryza sativa, Arabidopsis thaliana*, *Beta vulgaris*, *Glycine max* and *Helianthus annuus* Hsfs protein sequences that were downloaded from the UniProt database.

### Genetic structure, motifs composition and analysis of cis-acting elements

By studying the conserved motifs in FtHsf protein, the structural differences among different *FtHsf* genes were found (Additional file 2). We compared several protein sequences, and the exon-intron structures of the *FtHsf* genes were understood by comparing the predicted coding sequence with the corresponding full-length sequence by the Gene Structure Display Server online program. Eventually, we have known ten conserved motifs of the recognized Hsf proteins according to the MEME online program. Additionally, PlantCARE software (http://bioinformatics.psb.ugent.be/webtools/plantcare/html/?tdsourcetag=s_pcqq_aiomsg) was used to predict the cis-acting elements of 2000 bp upstream of all extended genes.

### Chromosomal distribution and gene duplication

We used Circos to process the chromosomal location information of the *FtHsf* genes. We made use of Multiple collinear scanning toolkits (MCScanX) to detect the gene replication events. The homology analysis maps of Tartary buckwheat were drawn up by the Dual Synteny Plotter software. And the homology relationships between the homologous *Hsf* genes and other varieties of Tartary buckwheat were revealed [34].

### Gene expression analysis

Firstly, the RNA of all samples was extracted with the EASYspin Plant RNAiso reagent (Aidlab, China). The cDNA was produced by 1 mg RNA sample with a Prime Script RT Reagent Kit with gDNA Eraser (TaKaRa) with SYBR Premix Ex Taq II (TaKaRa). Expression pattern of *FtHsf* genes identified in different tissues (stems, roots, leave, fruits and flowers) and fruits at three different stages (13, 19 and 25 DAP) from five strains of Tartary buckwheat were analyzed with qRT-PCR, and each Tartary buckwheat was analyzed three times [35]. The qRT-PCR primers of *FtHsf* genes listed in Additional file 4: Table S4 were obtained by Primer3 software (Additional file 4). We made the Tartary buckwheat H3 genes as the internal reference. The correlative expression data were calculated according to the 2^−(∆∆Ct)^ method [34].

### Subcellular localization

In order to verify the above subcellular localization prediction, we selected two *FtHsf* genes (*FtHsf18* and *FtHsf19*) as representatives to carry out subcellular localization experiments. First, the expression vectors of green fluorescent protein (GFP) tags were constructed [36], then the coding regions of *FtHsf18* and *FtHsf19* were amplified by PCR with specific primers and fused into the N-terminal of GFP under the control of the CaMV35S promoter. Finally, the subcellular localization of the GFP expression in *Arabidopsis* protoplasts was observed with the help of confocal microscope after 12 h of transformation [37].

### Statistical analysis

We processed and analyzed all the above data with the variance analysis with the Origin Pro 2018b statistics program and compared them by the least significant difference (LSD).

## Results

### Identification of the *FtHsf* genes in Tartary buckwheat

We used twice BLASTp methods to identify 29 *FtHsf* genes from the Tartary buckwheat genome after deleting redundant *FtHsf* genes because of the genome-wide shotgun strategy (Additional file 1). In this article, we renamed the *FtHsf* genes according to their chromosome locations, naming them from *FtHsf1 to FtHsf29* (Additional file 1).

We provided the gene characteristics including CDS, Mw, pI and subcellular localization. The 29 predicted FtHsf proteins ranged from 216 amino acids (FtHsf5*)* to 503 amino acids (FtHsf17). The Mw of the Hsf proteins ranged from 24.59 (FtHsf5*)* to 55.30 (FtHsf17) kDa, and the pI ranged from *4.77* (FtHsf5) to *9.1* (FtHsf6) (Additional file 1). The results subcellular localization showed that Hsf proteins were all situated in the nuclear (Additional file 1).

### Phylogenetic analysis and classification of the *FtHsf* genes

**Fig. 1:**
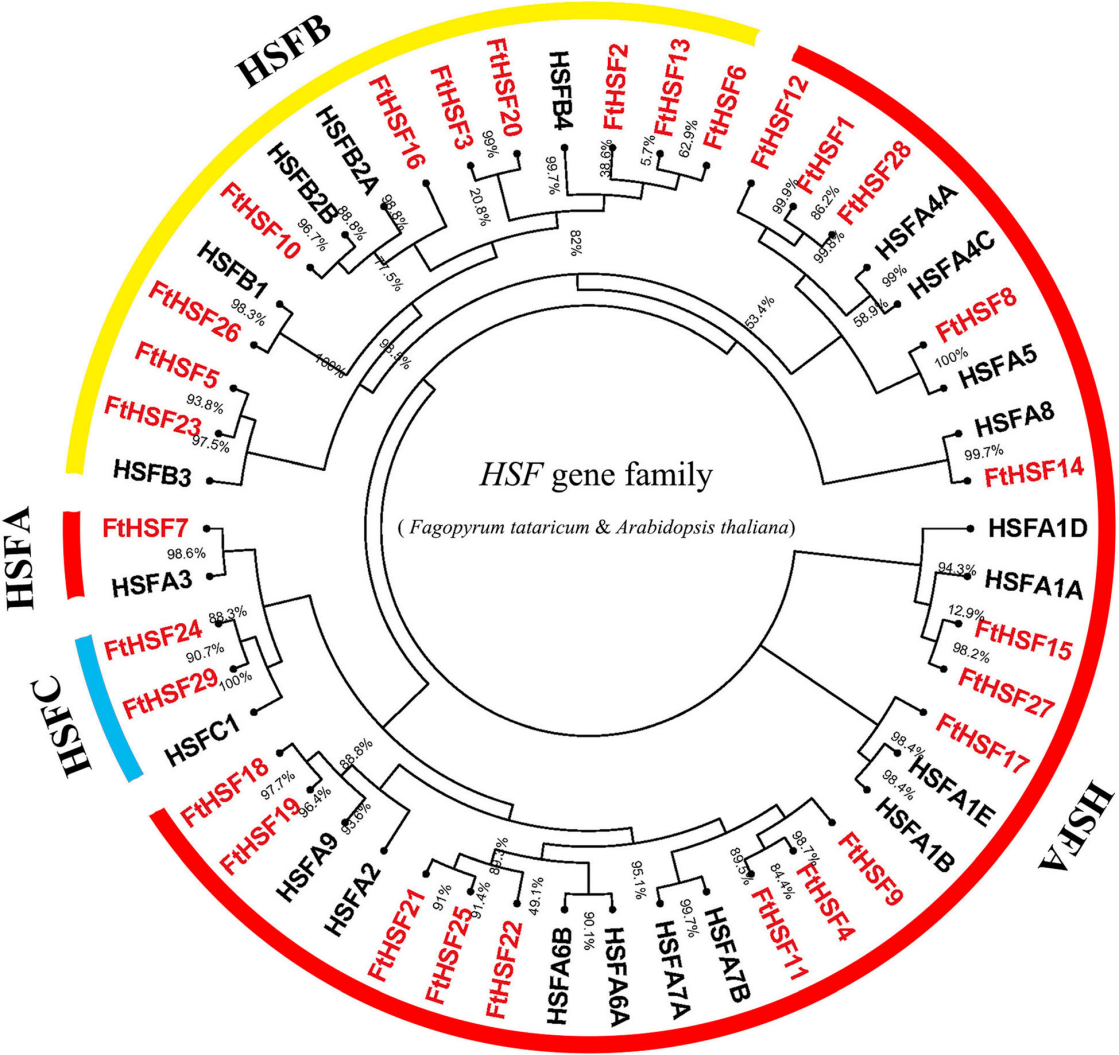
Unrooted phylogenetic tree representing the relationships among the *Hsf* genes of Tartary buckwheat and Arabidopsis. As shown in the figure, the phylogenetic tree is divided into 3 groups, including group A, B and C

**Fig. 2:**
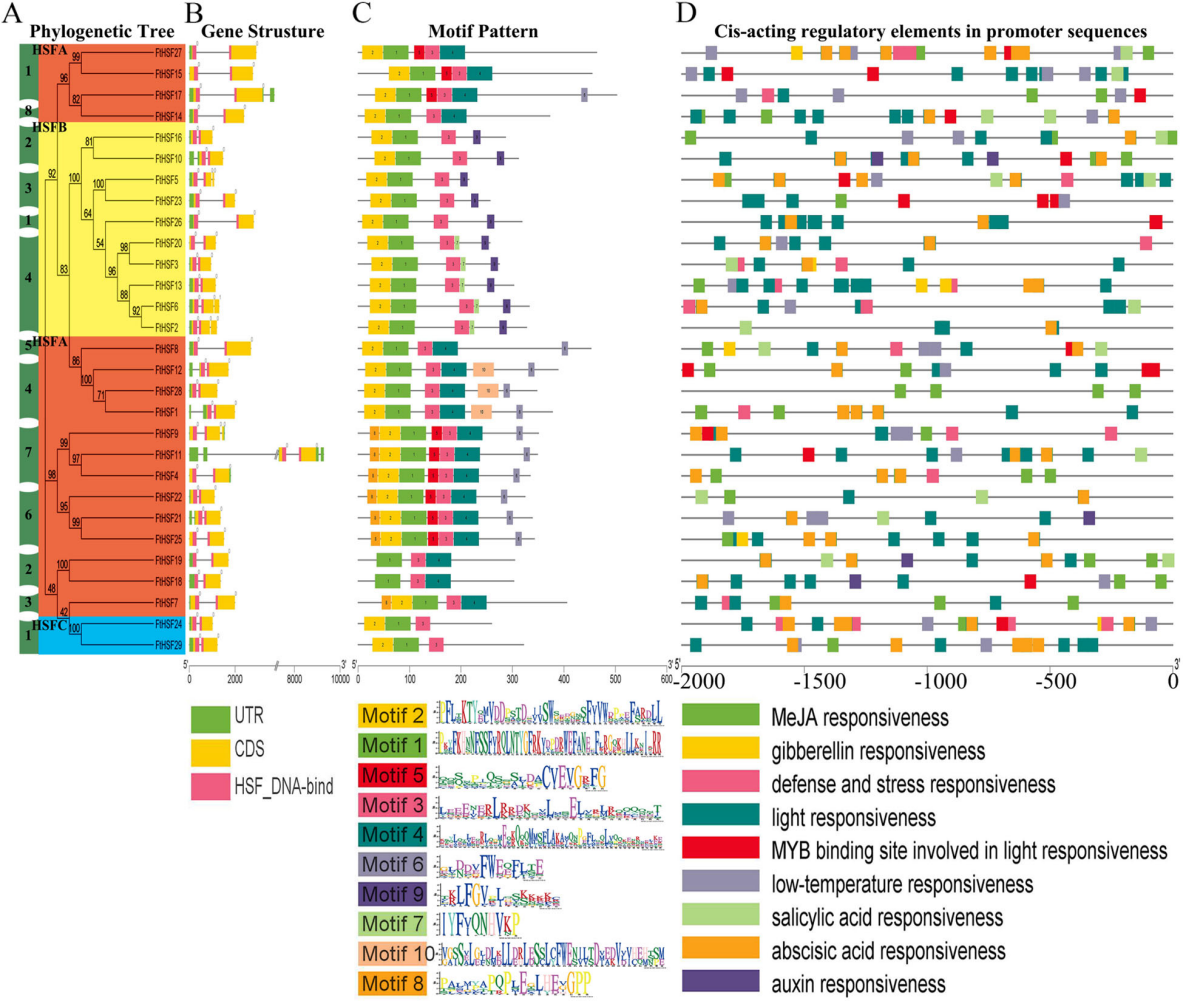
Phylogenetic relationships, gene structures, architecture of the conserved protein motifs and the cis-acting elements analysis of the *FtHsf* from Tartary buckwheat. **a** The phylogenetic tree was constructed based on the full-length sequences of Tartary buckwheat Hsf proteins using Geneious R11 software, including group A (A1, A2, A3, A4, A5, A6, A7, A8), group B (B1, B2, B3, B4) and group C (C1). **b** Exon-intron structures of Tartary buckwheat *Hsf* genes. Blue-green boxes indicate untranslated 5’- and 3’-regions; yellow boxes indicate exons; and black lines indicate introns. The *Hsf* domains are highlighted by pink boxes. The number indicates the phases of the corresponding introns. **c** The motif composition of the Tartary buckwheat Hsf proteins. The motifs, numbered 1–10, are displayed in different colored boxes. The sequence information for each motif is provided in Additional file 2. The length of the protein can be estimated using the scale at the bottom. **d** The cis-acting elements of the *FtHsf* promoter region, and different color blocks represent different elements

To investigate the phylogenetic relationship of the Tartary buckwheat Hsf proteins, we constructed a phylogenetic tree consisting of *Arabidopsis thaliana* (21 Hsf proteins) and Tartary buckwheat (29 Hsf proteins) (Fig. 1). According to the differences in the HR-A/B domain and phylogenetic relationships of FtHsf family members, the *FtHsf* genes were further divided into 3 big groups (named A, B and C) and 13 subfamilies, including A (A1, A2, A3, A4, A5, A6, A7, A8), B (B1, B2, B3, B4), and C1 (Figs. 1 and 2a). Tartary buckwheat is a dicotyledonous plant, and A9 and C2 only exist in monocotyledonous plants [22]. The B4 subfamily contained the largest number of FtHsf members, with five members. There were followed by A1, A4, A6 and A7 subgroups, all of which had three members of the FtHsf family. Then A2, B2, B3 and C1 subgroups all contained two members of the FtHsf family. Finally, A3, A5, A8 and B1 subgroups all contained only one member of the FtHsf family (Fig. 1).

### Gene structure, motif composition and cis-acting elements

In order to study the structural composition of *FtHsf* genes, we studied the exon and intron in detail including their amount and distribution (Fig. 2b). Gene structure analysis showed that the number of introns in different *FtHsf* genes was not the same. Most *FtHsf* genes only contained one intron, and four *FtHsf* genes (*FtHsf2*, *FtHsf5, FtHsf6 and FtHsf9*) contained two introns (Fig. 2b). The members of the same subfamily usually had similar exon / intron structures in terms of intron number and the exon length.

**Fig. 3:**
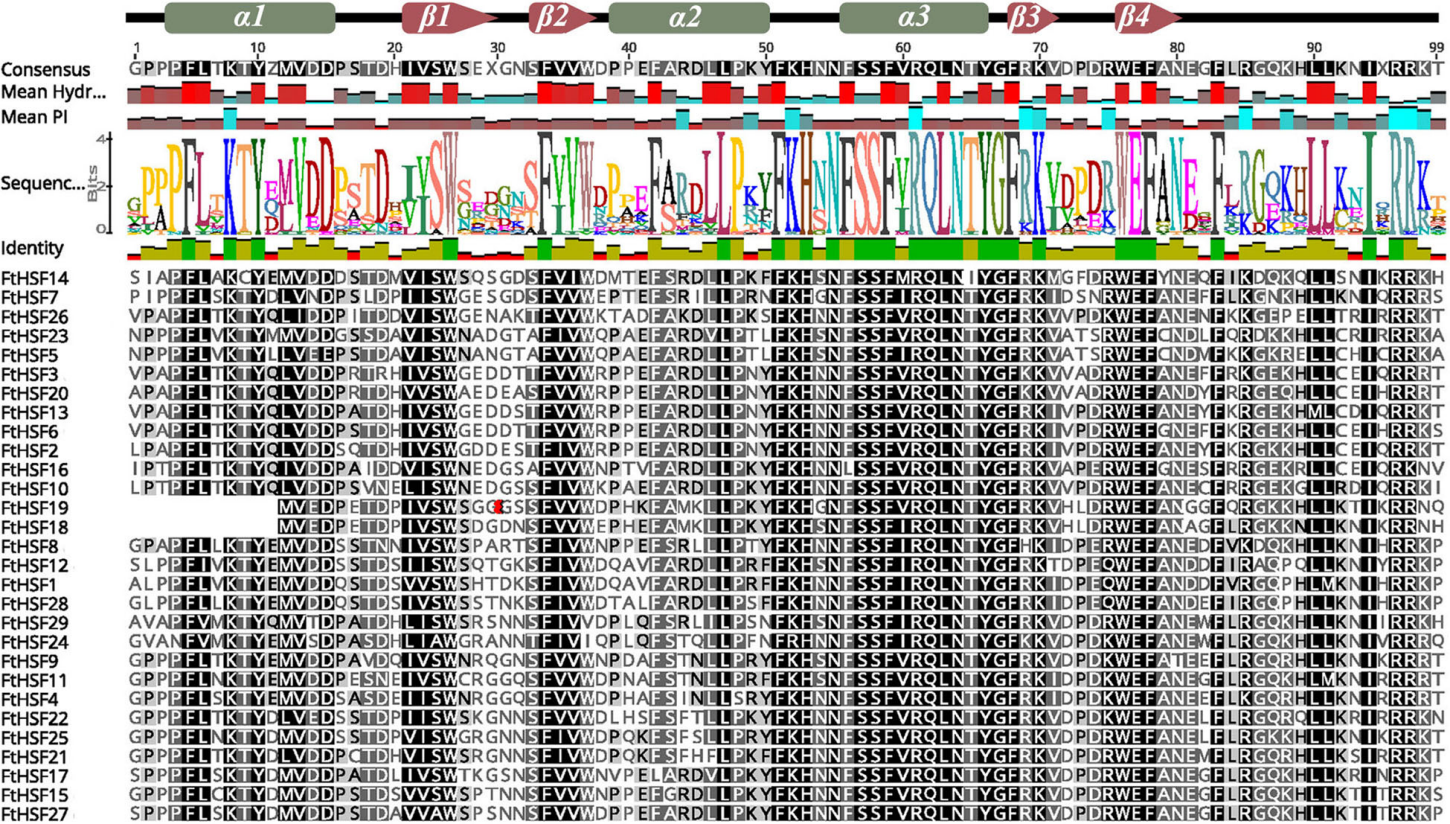
DBD domain sequences of FtHsfs identified by Pfam database were aligned by Clustal X 2.0 software and edited by DNAMAN software. The height of the color letter represented the conservative degree of the corresponding sequence, and the higher the letter, the more conservative it was. The helix-turn-helix motifs of DBD (α1-β1-β2-α2-α3-β3-β4) were shown at the top. Cylindrical tubes represented α1-helices and block arrows represent β-sheets

To further study the characteristic regions of the FtHsf proteins, the motifs of the Tartary buckwheat FtHsf proteins were analyzed by online MEME. According to the results of the MEME motif analysis, a schematic diagram was constructed to characterize the structures of the FtHsf proteins (Fig. 2c). According to the amino acid conserved sequences of the motifs 1, 2, 3, 4, 6, 9 and 10, they were divided into five categories (DBD, HR-A/B or OD, NLS, NES and AHA) (Fig. 2c, Additional file 2) [31]. It can be seen from the Fig. 3c that group A FtHsf members had the most conserved motifs, followed by group B and group C FtHsf members. Motifs 1 and 2 (DBD domain) were both found in 27 members of the FtHsf family, but only motif 1 was found in FtHsf18 and FtHsf19. The DBDs included 4 β rotation angles and 3 α helices in the N-terminal region (α1-β1-β2-α2-α3-β3-β4) (Fig. 3). And the helix motif (H2-T-H3) can specifically bind to the promoter of heat stress inducible gene, but the length of the DBD domain varies greatly [22]. The conserved motifs 3 and 4 after DBD domain were HR-A/B region, which was found in all members of the FtHsf family. Specially, we found the length of class A FtHsfs were longer than that of class B and class C FtHsfs (Fig. 2c, Additional file 2). And the reason for this is that all class A and class C FtHsf members have an expanded HR-A/B region [31]. The NLS domain contained conserved motifs 3 and 9, it existed in all members FtHsf family. However, only motif 3 was used to represent NLS domain in class A and class C, while NLS domain was represented by both motifs 3 and 9 in class B. The conserved motif 10 belongs to the NES region, but it only appeared in three Class A members (FtHsf1, FtHsf12 and FtHsf28) (Fig. 2c, Additional file 2). Therefore, all of 29 FtHsfs have NLS domain, but only three Class A members contain NES domain, and the two domains jointly maintain the balance of FtHsf inside and outside the nucleus [23, 31]. The conserved motif 6 was identified as a characteristic AHA domain, which is a structure that is unique to the group A family, while no AHA domain was found in group B or in group C (Fig. 2c, Additional file 2). Additionally, there are other conserved motifs in FtHsfs, but the action mechanism of these motifs is unclear. All in all, the conserved motif composition and the gene structure within the same group of FtHsf members were very similar, and the results of phylogenetic analysis supported the reliability of the population classification (Fig. 2, Additional file 2).

By analyzing the cis-acting elements in the promoter region, we found that most *FtHsf* genes contained multiple Light-responsive elements, ABA-responsive elements and MeJA-responsive elements. Nearly 50% of *FtHsf* genes contained Low-temperature responsive element, MYB-responsive element, Salicylic acid-responsive element and Defense and Stress responsive element, while only about 20% of *FtHsf* genes contained Auxin-responsive element and Gibberellin-responsive element (Fig. 2d). It can be inferred that *FtHsf* can not only participate in a variety of abiotic stress responses [38, 39], but also respond to a variety of exogenous hormones [40].

### Chromosomal distribution and homology analysis

**Fig. 4:**
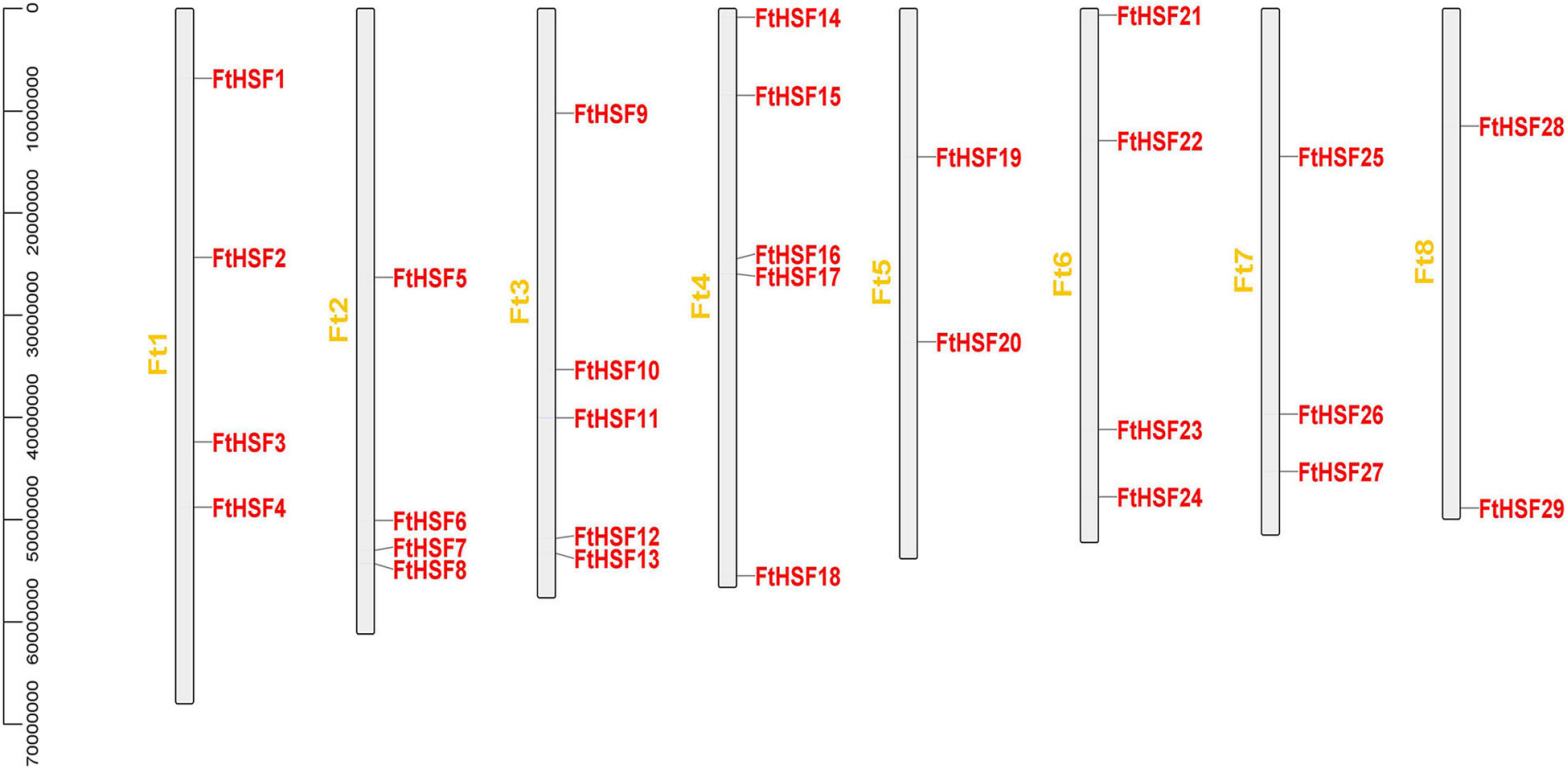
Schematic representations of the chromosomal distribution of the Tartary buckwheat *Hsf* genes. The number of the chromosome is shown on each chromosome

**Fig. 5:**
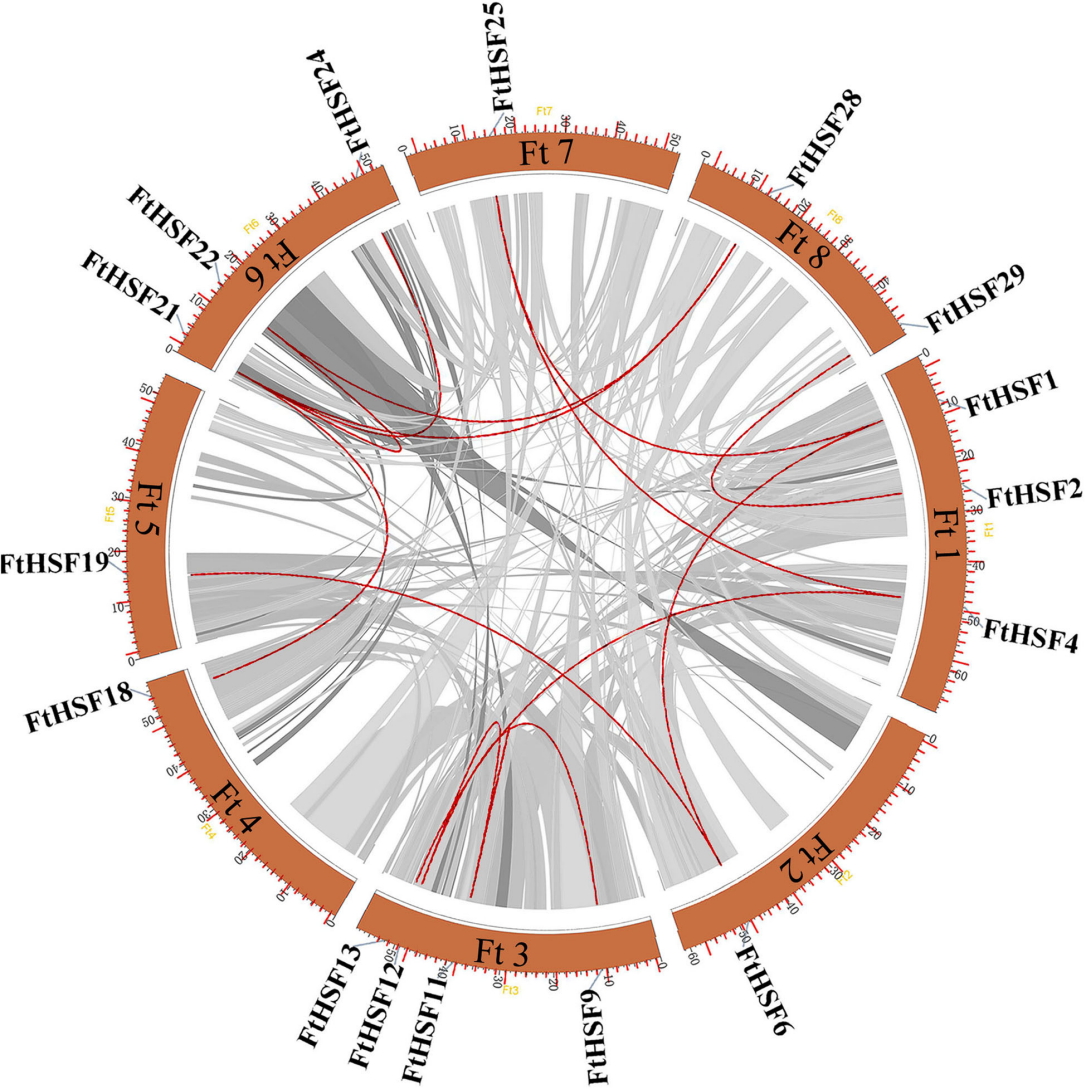
Schematic diagram of the relationship between different *FtHsf* genes on different chromosomes. Grey and red lines indicate all homology blocks in the Tartary buckwheat genome, and the red lines indicate duplicated *FtHsf* gene pairs

According to the study, there are eight chromosomes in Tartary buckwheat, and each chromosome has a different number of the *FtHsf* genes (Fig. 4). *FtHsf* genes were found in all chromosomes, among which the most *FtHsf* genes were found on chromosome 3 and chromosome 4, but chromosome 2 and chromosome 5 had only two *FtHsf* genes (Fig. 4). According to Holub, a chromosome region containing more than two genes within 200 kb is defined as a tandem duplication [41]. Homology analysis showed that there were no tandem duplication event sequences in the Tartary buckwheat (Fig. 5). Of the 29 *FtHsf* genes, 13 pairs of fragment duplication were found, with the most duplication events on chromosome 1 and chromosome 6 and only one on chromosome 4 and chromosome 5 (Fig. 5). These results showed that gene duplication may be the cause of the formation of some *FtHsf* genes and that these fragment duplication events were the main cause of *FtHsfs* evolution [42].

### Evolutionary and synteny analysis of the FtHsfs and the Hsfs of several different species

**Fig. 6:**
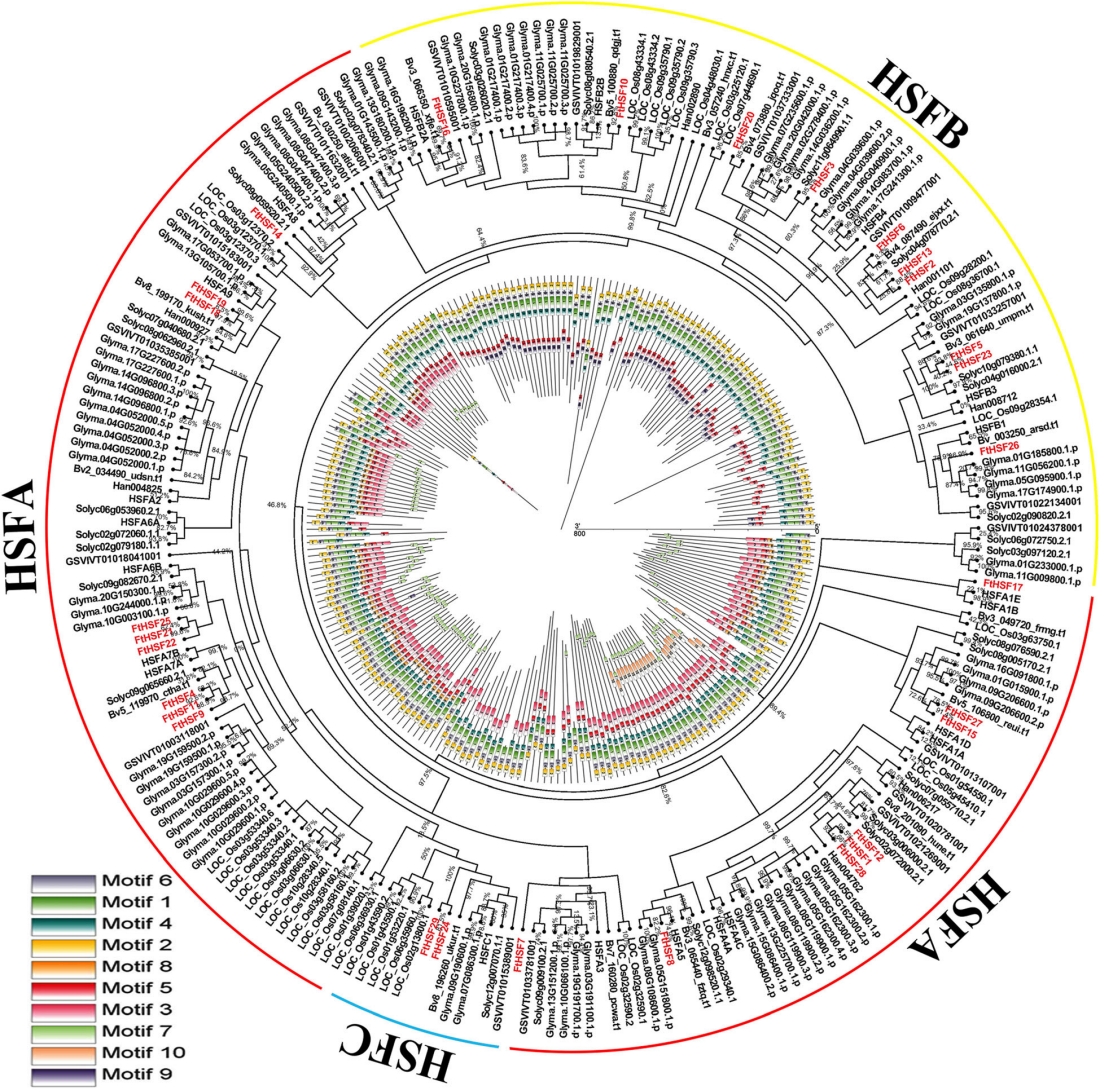
Phylogenetic relationships and motif compositions of the Hsf proteins from eight different plant species. Outside: an unrooted phylogenetic tree constructed using Geneious R11 with the neighbor-joining method. Inside: distribution of the conserved motifs in the Hsf proteins. The different colored boxes represent different motifs and their positions in each Hsf protein sequence

To further study the evolutionary relationship between the *FtHsf* genes, we used MEGA 5.0 to construct a phylogenetic tree that consisted of 8 representative species of Hsf protein sequences, including one monocotyledonous (*Oryza sativa*) and seven dicotyledonous plants (*Vitis vinifera*, *Solanum lycopersicum*, *Arabidops is thaliana*, *Beta vulgaris*, *Glycine max*, *Helianthus annuus* and *Fagopyrum tataricum*) (Fig. 6). According to the phylogenetic tree, Hsf members of the same subclass from different species gather together, and the Hsfs were divided into three big groups, named A, B and C (Fig. 6). Using MEME web servers, we searched the conserved motifs shared by the Hsf proteins. Finally, we obtained ten different conserved motifs and classified them according to their conservative sequence (Fig. 6, Additional file 2) [31]. Among which motif 1, motif 2, motif 4 and motif 6 encoded the *DBD* domain, motif 5 and motif 3 belonged to HR-A/B, and the motif 7 represented the AHA domain (Fig. 6, Additional file 2). Almost all Hsf families have motif 1, 2, 4 and 6, motif 3 and 5, indicating that DBD domain and HR-A/B domain were very conservative in Hsf families (Fig. 6). Motif 7 only existed in some members of Class A Hsf family (Fig. 6), the AHA region was the key area for Hsfs to play a self-activating role, and it was speculated that the mechanism of Hsfs self-activation was similar in different plants [21, 23]. As shown in Fig. 5, the Hsfs of the same subclass in different species usually had the same motifs composition (such as FtHsf3 and Solyc11g064990.1.1), it was speculated that there may be similar functions between proteins.

**Fig. 7:**
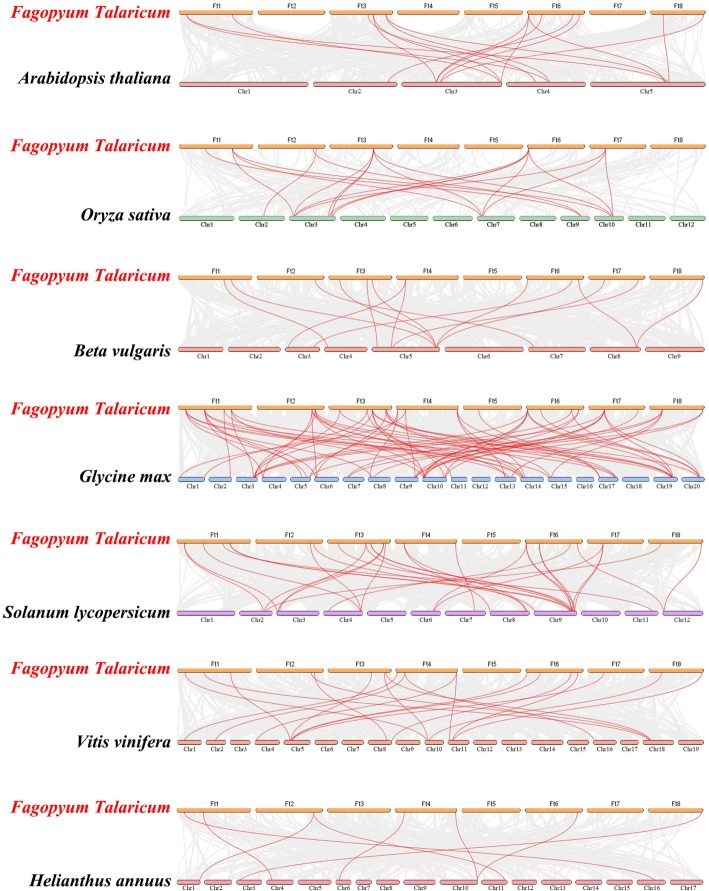
Synteny analysis between the *Hsf* genes of Tartary buckwheat and seven representative plant species. Gray lines in the background indicate the collinear blocks within Tartary buckwheat and other plant genomes, while the red lines highlight the syntenic *Hsf* gene pairs

To understand more about the phylogeny of Tartary buckwheat *FtHsf* genes family, the *Hsf* gene of the Tartary buckwheat was subjected to a synteny analysis with the *Hsf* gene of the other seven typical plants, including six dicotyledonous plants (*Arabidopsis thaliana, Beta vulgaris, Glycine max, Helianthus annuus, Solanum lycopersicum, and Vitis vinifera*) and a monocotyledonous plant (*Oryza sativa*) (Fig. 7). There were 23 *FtHsf* genes that were synchronized with those in *Glycine max*, and then there was *Solanum lycopersicum* (20), *Vitis vinifera* (18), *Beta vulgaris* (13), *Arabidopsis thaliana* (11), *Helianthus annuus* (7), and *Oryza sativa* (7) (Fig. 7, Additional file 3). The number of homologous pairings of the other 6 species (*Glycine max*, *Solanum lycopersicum*, *Vitis vinifera*, *Oryza sativa, Arabidopsis thaliana*, *Beta vulgaris* and *Helianthus annuus*) were 67, 31, 20, 19, 16, 14, and 8 (Fig. 7, Additional file 3). The results showed that the genetic relationship between Tartary buckwheat *Hsf* genes and soybean *Hsf* genes was close. At the same time, we could find that some *FtHsf* genes were associated with multiple *Hsf* genes in other species, for example, the *FtHsf11* of buckwheat was associated with five *Hsf* genes in soybean and the rice, respectively (Fig. 7, Additional file 3). The *FtHsf11* may play a significant role in the evolution of the *FtHsf* gene family.

### Expression patterns of *FtHsf* genes in different plant tissues

**Fig. 8:**
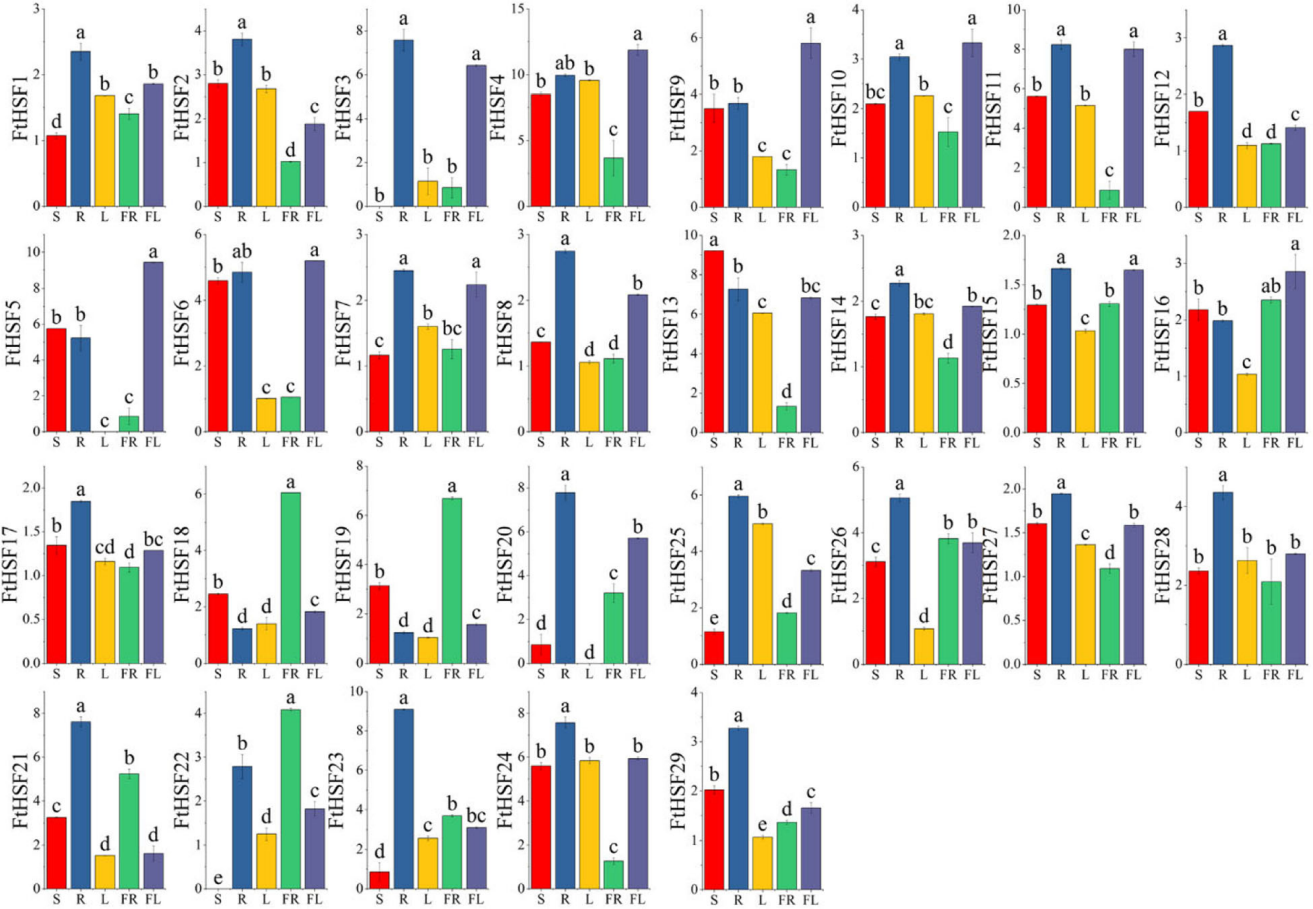
Tissue-specific gene expression of 29 Tartary buckwheat *Hsf* genes. The expression patterns of the 29 Tartary buckwheat *Hsf* genes in the flower, leaf, root, stem and fruit tissues were examined by qRT-PCR. Error bars were obtained from three measurements. Lowercase letter(s) above the bars indicate significant differences (α = 0.05, LSD) among the treatments

**Fig. 9:**
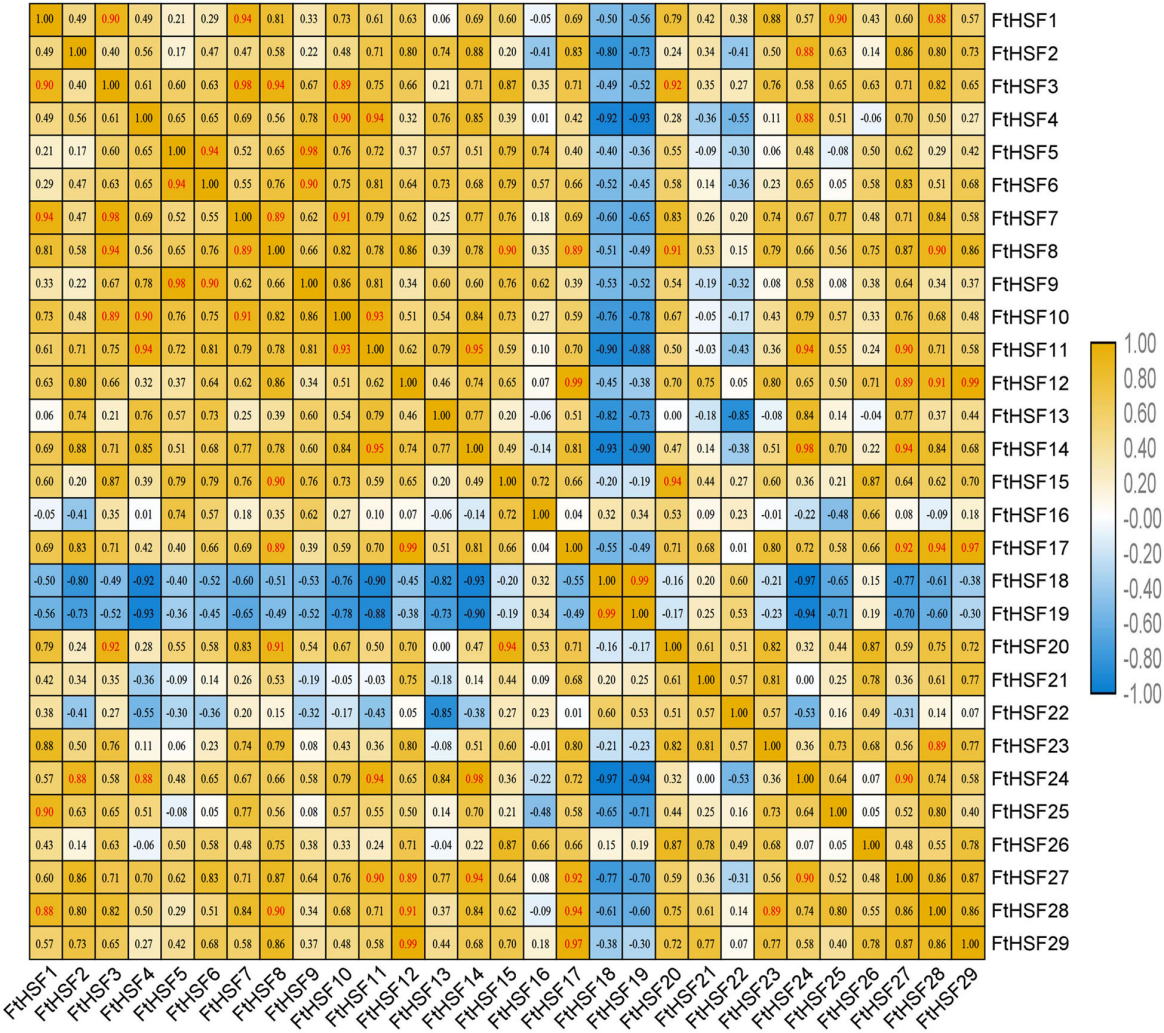
The correlations between the gene expression patterns of *FtHsfs*. Blue: positively correlated; Purple: negatively correlated. * indicates a significant correlation at the 0.05 level

**Fig. 10:**
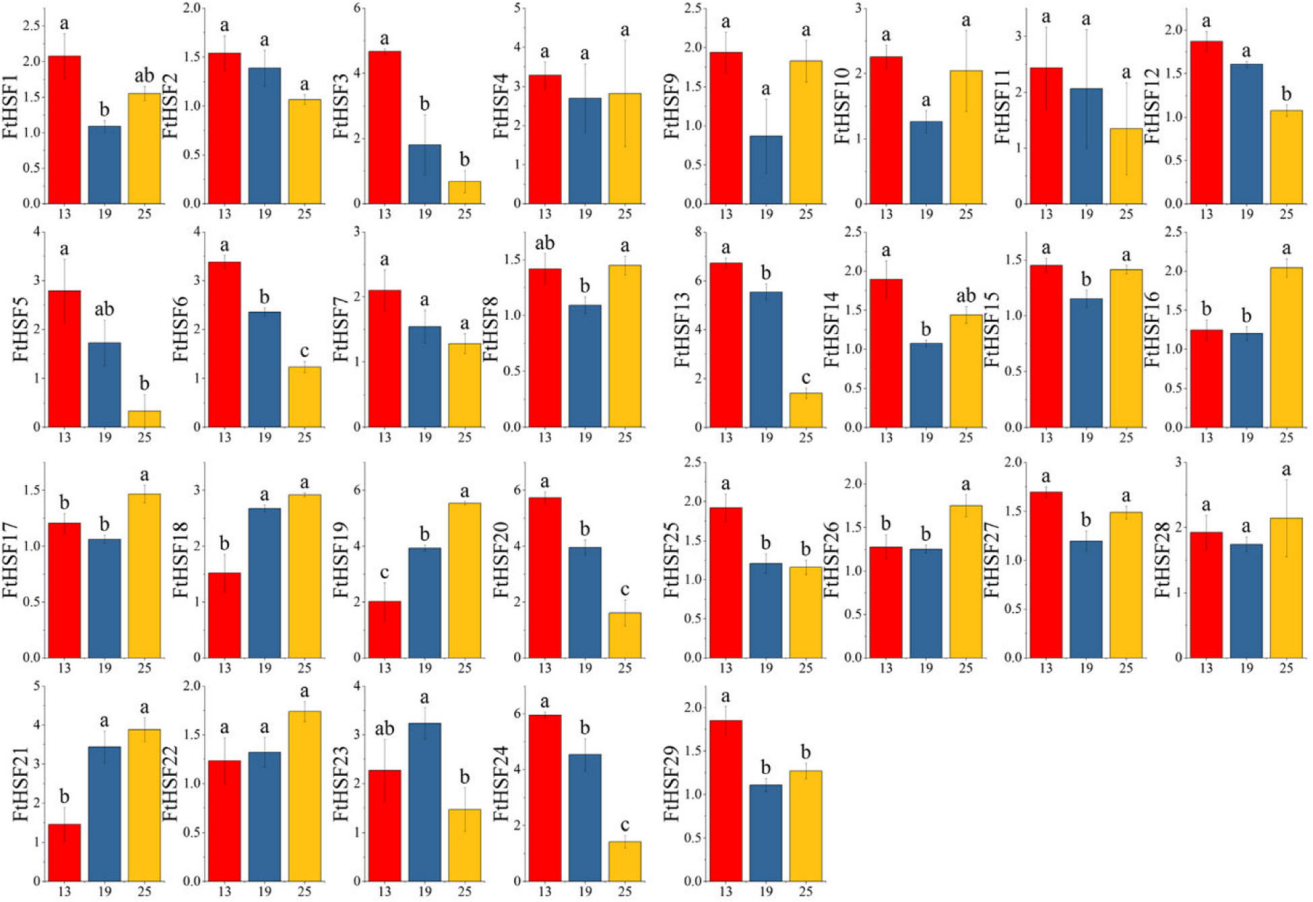
The gene expression patterns in buckwheat fruit at different developmental stages (13, 19, and 25 days after pollination, DAP). The expression patterns in buckwheat fruit t at different developmental stages were examined by qRT-PCR. Error bars were obtained from three measurements. Lowercase letter(s) above the bars indicate significant differences (α = 0.05, LSD) among the treatments

The qRT-PCR was used to determine the expression of 29 *FtHsf* genes in different tissues and the physiological functions of *FtHsf* genes were discussed. (Fig. 8). The results showed that there were significant differences in the expression of the *FtHsf* genes in different tissues/organ, showing that the *FtHsfs* had a variety of functions in the growth and development of Tartary buckwheat. Some *FtHsf* genes had prominent expression in Tartary Buckwheat tissues/organ. Three *FtHsf* genes (*FtHsf18/FtHsf19/FtHsf22*) were highly expressed in fruit (Fig. 8). Seven *FtHsf* genes (*FtHsf10/FtHsf9/FtHsf6/FtHsf15/FtHsf4/FtHsf16/FtHsf5*) were high expression in the flowers than in the other tissues/organs. According to the study, we could find that many *FtHsf* genes were highly expressed in the leaves (except *FtHsf20/FtHsf5*) (Fig. 8). The majority of the *FtHsf* genes were expressed in Tartary buckwheat stems except *FtHsf3*. Besides, we also studied the correlations among the *FtHsf* genes expression patterns in the roots, stems, flowers, leaves and fruit of Tartary buckwheat (Fig. 9). The results showed that many *FtHsf* genes belonged to the positive correlation, and it was worth noting that there was a significant positive correlation between some *FtHsf* genes, for example, *FtHsf18* and *FtHsf19*, *FtHsf12* and *FtHsf29*, *FtHsf5* and *FtHsf9* and so on (Fig. 10).

### Differential expression of the *FtHsf* genes during fruit development in Tartary buckwheat

**Fig. 11:**
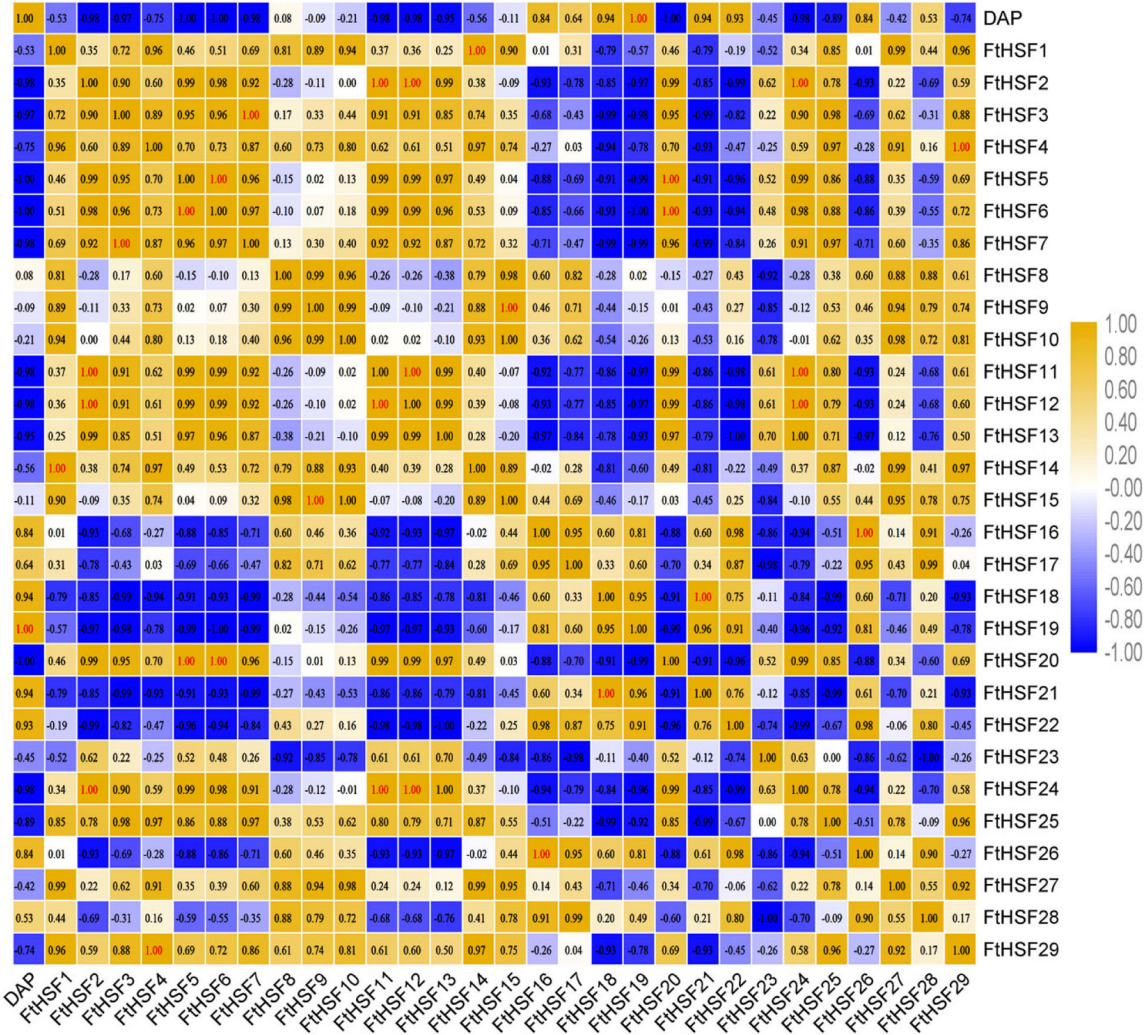
The correlations between the *FtHsfs* genes expression patterns of buckwheat fruit at different developmental stages. Orange: positively correlated; Green: negatively correlated. The red numbers indicate a significant correlation at the 0.05 level

To explore the possible role of the *FtHsf* genes in different stages of Tartary buckwheat fruit development, we compared the gene expression patterns at different buckwheat fruit development stages (13, 19, and 25 days after pollination, DAP) (Fig. 10) [25]. As the results show, all members of the *FtHsf* family were expressed at the whole stage of buckwheat fruit development, and the expression of most of the *FtHsf* genes has changed in these three stages (Fig. 10). The expression of some *FtHsfs* showed obvious trend in different developmental stages. With the maturity of the Tartary buckwheat fruit, the expressions of 14 *FtHsfs* were fluctuating up and down, the expression level of 11 *FtHsfs* decreased, and only 4 *FtHsfs* (*FtHsf18, FtHsf19, FtHsf21* and *FtHsf22*) expression were on the rise (Fig. 10). It was speculated that these four *FtHsf* genes play a certain role in the ripening process of Tartary buckwheat fruits. At the same time, by studying the correlation between *FtHsf* genes and fruit development, and the correlation between *FtHsf* genes in the process of fruit development (Fig. 11), we found that the expression of most genes was negatively correlated with fruit development, and only the *FtHsf19* gene showed significant positively correlated. At the same time, we found that most *FtHsf* genes were positively correlated with each other during fruit development of Tartary buckwheat, and there was a significant positive correlation between some of these genes (*FtHsf18* and *FtHsf21, FtHsf3* and *FtHsf7, FtHsf1* and *FtHsf14* and so on) (Fig. 11).

### FtHsf18 and FtHsf19 were localized in the nucleus

**Fig. 12:**
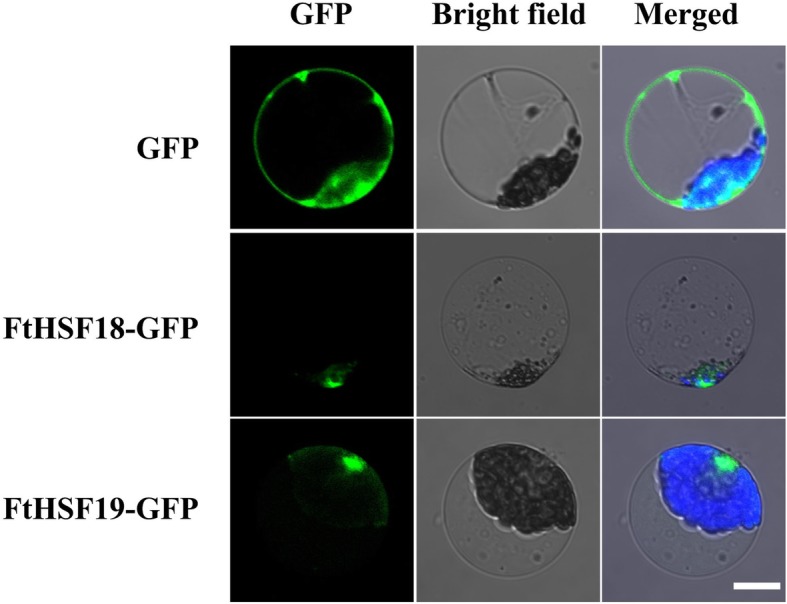
Subcellular localization of FtHsf18 and FtHsf19 in *Arabidopsis* protoplasts. GFP and FtHsf18/19-GFP under the control of the CaMV35S promoter separately transiently expressed in *Arabidopsis* protoplasts

In order to verify the prediction of subcellular localization, two *FtHsf* genes (*FtHsf18* and *FtHsf19*) were selected as representatives to carry out the experiment (Fig. 12). Subcellular localization analysis was carried out by constructing the expression vector labeled with green fluorescent protein (GFP) [36], the expression of GFP in protoplasts vector of mesophyte cells of *Arabidopsis thaliana* was used as the control group, and the subcellular localization of GFP expression was observed by confocal microscope [37]. It can be seen from Fig. 12 that the distribution of GFP in protoplasts of the control group was uniform, while the FtHsf18 and FtHsf19 fusion proteins were only located in the nucleus. This showed that FtHsf18 and FtHsf19 proteins were located in the nucleus, and the prediction of subcellular localization was correct.

## Discussion

### *FtHsf* genes identification and evolutionary analysis in Tartary buckwheat

*Hsf* genes are the heat stress transcription factors [31]. The number and motif composition of *Hsf* genes are often different in different species. It was reported that 28, 21, 19, 18,16 and 13 *Hsf* genes were found in poplar [24], Arabidopsis [21], grapes [43], tomatoes [31], alfalfa [24] and beets [44], respectively. In this research, we found 29 *Hsf* genes in the Tartary buckwheat, and all the FtHsf proteins were located in the nucleus (Additional file 1, Fig. 12). Compared with other dicotyledonous plants, more *Hsf* genes were found in Tartary buckwheat genome. The recombination and expansion of the genome can usually alter the number of members of a gene family [25]. The genomic replication events which lead to the duplication of the gene family often occur during the evolution of angiosperms [25]. It was presumed that there were more genomic replication events in Tartary buckwheat after differentiation from the early ancestors of other species.

Different gene duplication patterns contribute to the amplification of corresponding gene families in plant genomes, such as genome duplication, tandem duplication and fragment duplication [45]. It is reported that some large gene families (such as WRKY families) are more likely to be amplified by fragment duplication and tandem duplication than by other replication events [46]. However, gene families such as MADS and NBS expand primarily through transposed duplications. Gene replication causes the emergence of more than 90% regulatory genes in *Arabidopsis thaliana* [47]. In this article, the synteny analysis confirmed that the expansion of the *FtHsf* gene family in Tartary buckwheat mainly originated from fragment duplication, not from tandem duplication (Fig. 5) [48, 49]. *FtHsf21* and *FtHsf22* belong to a pair of gene pairs duplications by fragments (Fig. 5). By studying their expression patterns, it was found that *FtHsf21* was highly expressed in the roots, but *FtHsf22* was highly expressed in the fruits (Fig. 8). Therefore, we could know that the specific expression of duplication genes was different. However, through the comparison of their motifs, it was found that their motifs were the same (Fig. 2). It was speculated that the reason for the differential expression of these genes may be due to a gene mutation during gene duplication, which resulted in the loss of function of some parts of the gene [50] (Additional file 2).

*Fagopyrum tataricum, Arabidopsis thaliana, Beta vulgaris, Glycine max, Helianthus annuus, Solanum lycopersicum,* and *Vitis vinifera* are dicotyledonous plants, and *Oryza sativa* is a monocotyledon plant. Phylogenetic tree analysis showed that Hsf in the same subgroup had similar motif composition, and contained both monocotyledonous and dicotyledonous plants in most subgroups (Fig. 6). It was speculated that *Hsf* genes appeared in monocotyledonous and dicotyledonous plants before differentiation [50, 51]. Synteny analysis showed that 19 pairs of homologous genes between Tartary buckwheat and rice were more than those between Tartary buckwheat and *Arabidopsis thaliana*, sugar beet and sunflower (Fig. 7, Additional file 3). This phenomenon indicates that there is no significant difference between dicotyledonous plants and monocotyledonous plants, which further indicated that the *Hsf* family appeared before the differentiation of monocotyledonous plants and dicotyledonous plants [50, 51].

### Functional analysis of conserved domains of *FtHsf* genes in Tartary buckwheat

*Hsf* is dependent on NLS transport into the nucleus [52]. The NLS is located at the C-terminus of the HR-A/B region and the NLS is generally arginine-rich (R) and lysine-rich (K) region [53]. NES is on the C-terminal side of *Hsf*, and NES plays a role in the extranuclear transport of *Hsf* [53]. The NLS and NES maintain the balance of *Hsf* in and out of the nuclear system. All FtHsfs contained the NLS domain, but only 3 FtHsrfs (FtHsf1/12/18) had an NES domain (Fig. 2, Additional file 2). Therefore, all members of the Tartary buckwheat *FtHsf* family can play a role in the nucleus, and some of them can also travel inside and outside of the nucleus under certain conditions. The AHA motif was one of the characteristic structures of group A FtHsfs, and the transcriptional activation activities of group A FtHsfs were worked by the AHA of the C-terminal activation region. The AHA region is rich in aromatics, hydrophobic and acidic amino acid residues [54]. In the Tartary buckwheat Hsf family, there were 11 FtHsf members of class A with AHA motifs, and neither class B nor class C members contained AHA domain (Fig. 2). It was predicted that class A members with AHA domain have self-transcriptional activation activity [55, 56]. As a result, other FtHsf members with no AHA structure didn’t have transcriptional activation activities themselves, so they cannot exercise transcriptional activation alone but by forming a heteropolymer by binding to class A FtHsf to perform their functions [55, 56]. Thus, it can be predicted that class A FtHsf members with an AHA structure play an important role in Tartary buckwheat response to an environment with high temperatures [21, 22].

### Tartary buckwheat *Hsf* genes may play an important role in plant development

By analyzing the cis-acting elements in the promoter region of *FtHsf* genes, we found that the promoter region of *FtHsf* genes include not only Light-responsive elements and Low-temperature responsive element, but also components such as ABA-responsive elements, MeJA-responsive elements and MYB-responsive elements and so on (Fig. 2d). Which means that *FtHsf* genes can be involved not only in various stress responses, but also in the regulation of the growth and development of Tartary buckwheat [57]. *FtHsf5* was significantly expressed in Tartary buckwheat flowers, suggesting that *FtHsf5* may be involved in the development of Tartary buckwheat flowers. In a multi-species phylogenetic tree, genes in a branch usually have similar functions [50]. In Fig. 9, we found two special genes (*FtHsf20* and *FtHsf3*) from the *FtHsf* genes which have a significant expression in the roots, and *FtHsf20* and *FtHsf3* were expressed in only three kinds of tissues. At the same time, we found that their expression showed a high positive correlation (Fig. 9). It was interesting that they belonged to the same subgroup (B4) and were closely related to the *AtHsfB4* (*AT1G46264.1*) gene in *Arabidopsis thaliana* (Fig. 1). It was worth noting that the overexpression of *AtHsfB4* gene in *Arabidopsis thaliana* led to the shortening of plant root length [32], so we speculated that *FtHsf20* and *FtHsf3* can also be involved in regulating the length of Tartary buckwheat root.

Meanwhile, we found that the expression levels of both *FtHsf18* and *FtHsf19* genes were the highest in the fruit (Fig. 8), and there was a significant positive correlation between them through Fig. 10. At the same time, by comparing the expression patterns of the *FtHsf* genes at different buckwheat fruit development stages (13, 19, and 25 days after pollination, DAP), founding that the expression of *FtHsf18* and *FtHsf19* increased gradually at the later stage of fruit development (Fig. 10). In addition, they also showed a high positive correlation in the fruit development of Tartary buckwheat (Fig. 11). From the phylogenetic tree of Tartary buckwheat and *Arabidopsis thaliana* (Fig. 2), we found a close relationship between *FtHsf18/FtHsf19* and *AtHsfA9 (AT5G54070)* in Arabidopsis. *AtHsfA9* gene in *Arabidopsis thaliana* is regulated by the transcription factor of acid-insensitive 3 (ABI3) and participates in the regulation of fruit maturation, the higher the content of ABA in fruit, the higher the expression of *AtHsfA9* gene [58]. The expression patterns of *AtHsfA9* in the different tissues, and at different stages of fruit development were similar to those of *FtHsf18* and *FtHsf19* [5]. Meanwhile, the content of ABA during the three stages of Tartary buckwheat fruit development (13, 19, and 25 days after pollination, DAP) increased gradually, while the expression of *FtHsf18* and *FtHsf19* during the three stages of buckwheat fruit development also increased gradually (Fig. 10) [25, 34, 58]. Therefore, we speculated that the *FtHsf18* and *FtHsf19*genes of Tartary buckwheat may also be regulated by ABI3 and participated in the regulation of fruit ripening.

## Conclusions

Overall, in this study, we found that the numbers, chromosomal locations, protein functional domains and expression patterns of 29 Tartary buckwheat *FtHsf* family genes are diverse and that they may be important for the plant to regulate the responses to abiotic stress and growth and development. This preliminary study on the *Hsf* genes of Tartary buckwheat provides a basis for further analyzing the function of the genes in different groups and for improving the stress resistance and yield of crops by using the related characteristics of the *Hsf* genes.

## Supplementary information

**Additional file 1. **List of the 29 *FtHsf* genes identified in this study.

**Additional file 2.** Analysis and distribution of conserved motifs in Tartary buckwheat and other plants Hsf proteins.

**Additional file 3.** One-to-one orthologous relationships between Tartary buckwheat and other seven plant species.

**Additional file 4.** Primers of sequences.

## Data Availability

The genome sequences of Tartary buckwheat used for identifying the *FtHsf* genes in this study were located in the Tartary Buckwheat Genome Project (TBGP; http://www.mbkbase.org/Pinku1/). The Tartary buckwheat accession (XIQIAO) materials used in the experiment were supplied by Professor Wang Anhu of Xichang University. All the datasets supporting the conclusions of this study are included in this article and its Additional files.

## Abbreviations

AA: Amino acid
ABI3: Abscisic acid-insensitive3
AHA: An activator motif
DAP: Days after postanthesis
DBD: N-terminal DNA-binding domain
GFP: Green fluorescent protein
GSDS: Gene structure display server
HMM: The hidden Markov model
HR-A/B: Composed of heptad repeats of hydrophobic amino acid residues
HSE: Heatstress element
Hsfs: Heat shock transcription factors
Hsps: Heat shock proteins
K: Lysine
LGs: Linkage groups
LSD: The least significant difference test
MADS: Minichromosome Maintenance1, Agamous, Deficiens and Serum Response Factir
Mw: Molecular weight
NBS-LRR: Nucleotide-binding site-leucine-rich repeat
NES: A nuclear export signal region
NJ: The neighbor-joining
NLS: A nuclear localization signal region
OD: An adjacent oligomerization domain
pI: Isoelectric point
qRT-PCR: Quantitative real-time polymerase chain reaction
R: Arginine
TBGP: Tartary buckwheat genome project
